# The Prevalence of Hearing Symptoms Associated With Patulous Eustachian Tube Dysfunction Following Bariatric Surgery at King Khalid University Hospital, Saudi Arabia

**DOI:** 10.7759/cureus.43255

**Published:** 2023-08-10

**Authors:** Khalid A Albawardi, Faisal A Alsanad, Hamdan S Aldosari, Sarah A Alhelal, Mubashir M Alasmari, Mada A Alsadi, Abdullah Aldohayan

**Affiliations:** 1 Medical School, King Saud University, Riyadh, SAU; 2 Otolaryngology - Head and Neck Surgery, King Saud Bin Abdulaziz University for Health Sciences, Riyadh, SAU; 3 Department of Surgery, College of Medicine, King Saud University, Riyadh, SAU

**Keywords:** patulous eustachian tube, pet, loss of weight, eustachian tube pathology, bariatric surgery complications

## Abstract

Background

A patulous Eustachian tube (PET) is defined as a persistent tubal opening, which affects 0.3-6.6% of the population, with a female preponderance. PET is caused by the loss of subcutaneous adipose tissue enclosing the cartilaginous portion of the Eustachian tube (Ostmann’s pad) as a result of acute, rapid, and substantial loss of weight which occurs during bariatric surgery. The most common complaint of PET is autophony, in which patients hear their own voices or breaths, with additional symptoms including crackling sounds, tinnitus, and aural congestion. In this study, we aimed to determine the prevalence of symptoms associated with PET dysfunction subsequent to bariatric surgery at King Khalid University Hospital (KKUH).

Methodology

A cross-sectional study was conducted at KKUH, Saudi Arabia. The presence of symptoms consistent with the diagnosis of PET dysfunction was assessed through in-person and telephonic interviews of bariatric surgery patients using a standardized questionnaire.

Results

A total of 450 patients were evaluated. The preoperative body mass index (BMI) of evaluated patients ranged from 28 to 117 kg/m^2^ (mean = 46.07 kg/m^2^), with no significant difference between symptomatic and asymptomatic groups (p = 0.303). The postoperative BMI ranged from 16 to 100 kg/m^2^ (mean = 29.37 kg/m^2^), with no significant difference between symptomatic and asymptomatic groups (p = 0.263). Hypertension was the most prevalent comorbid condition (12.2%), followed by diabetes (9.3%). In total, 91 (20.22%) patients exhibited symptoms (aural fullness and autophony) compatible with patent auditory tube dysfunction.

Conclusions

Overall, 20.22% of the bariatric surgery patients in our study sample displayed symptoms consistent with patulous auditory tubal dysfunction. The preoperative and postoperative BMI of symptomatic and asymptomatic patients did not differ significantly. To improve patient outcomes and satisfaction following bariatric surgery hearing symptoms associated with PET need to be included in postoperative follow-ups after bariatric surgery.

## Introduction

The Eustachian tube is a dynamic, thin, epithelial-lined conduit that connects the middle ear to the lateral wall of the nasopharynx. The medial two-thirds of the tube are fibrocartilaginous and the lateral one-third is bony [1]. It transmits sound waves from the tympanic membrane to the cochlea [1]. It is closed at repose, providing the middle ear with balance, structure, and protection from nasopharyngeal secretions. It only opens during gnawing, yawning, and sneezing with the assistance of the salpingopharyngeus, tensor tympani, levator, and tensor veli palatini muscles [2]. On the other hand, Ostmann’s fat pad, a cone-shaped adipose fatty tissue located on the inferomedial aspect of the cartilaginous part, ensures tube closure and inhibits the reflux of secretions [2]. The Eustachian tube serves multiple purposes, including drainage of fluids, protection of the middle ear from excessive noise or pathogens, ventilation, and equalization of intratympanic pressure with the external auditory canal. The inability to conduct the aforementioned actions is referred to as Eustachian tube dysfunction [3].

A patulous Eustachian tube (PET) is defined as a persistent tubal opening [4]. Therefore, aberrant transmission of sound from the nasopharyngeal cavity to the ears results in aural congestion, autophony, and a crackling sound in the hearing. It affects 0.3-6.6% of the population, with a female preponderance [4]. Weight loss is the most frequently cited risk factor for PET [5]. Pregnancy, radiation therapy, neurological disorders, and diphtheria are additional risk factors [6]. Furthermore, it can be a complication of surgical procedures such as cleft palate repair and adenoidectomy [6]. Over the past several decades, the global prevalence of obesity has consistently risen [7]. Educational, pharmacological, surgical, and behavioral interventions have been employed to combat this global burden [7]. According to the criteria of the National Institutes of Health, patients with a body mass index (BMI) >40 kg/m^2^ and >35 kg/m^2^ are candidates for surgical intervention (bariatric surgery) [8]. After bariatric surgery, the prevalence of eustachian tube patulosis is higher than in the general population [5]. Moreover, bariatric surgery in patients with anorexia and inadequate nutrition may predispose them to tubal permeability due to the loss of subcutaneous peritubal adipose tissue that surrounds the Eustachian tube (Ostmann’s fat pad) following rapid and substantial weight loss [5].

PET is diagnosed clinically based on the presence of risk factors, symptoms, and evidence of tympanic membrane excursions during deep respiration, as measured by otoscopy or tempanometry [9]. Unless another pathology is suspected, radiological tests are not routinely used to diagnose PET dysfunction [9]. The most prevalent complaint of PET is autophony, with patients describing it as eating difficulties due to the transmission of chewing sounds from the nasal cavity to the ears, or the perception of loud voices in normal dialogues. Additionally, symptoms include crackling sounds, tinnitus, and aural congestion [4]. Symptoms improve in the supine position and deteriorate with excessive conversation or when decongestants are used [4]. Clinical signs and symptoms can be used to diagnose Eustachian tube dysfunction, according to the medical literature. This study sought to determine the prevalence of signs and symptoms associated with PET dysfunction in bariatric surgery patients at King Khalid University Hospital (KKUH).

## Materials and methods

This is a cross-sectional study conducted at KKUH between 2022 and 2023. The study was authorized by King Khalid University Hospital’s ethical committee. At the hospital affiliated with King Khalid University, 450 patients undergoing bariatric surgery were recruited from three BMI levels, each of which included 150 patients (30-39.9-40-49.9-50). Every participant provided informed consent. The study included patients with at least six months of postoperative follow-up at KKUH regardless of gender. Those with a history of auditory tubal dysfunction, previous otological or nasal procedures, and those who underwent bariatric surgery outside of KKUH were excluded. All patients were interviewed using a standardized questionnaire to determine the presence of symptoms compatible with the patent auditory tube. Affirmative questionnaire responses regarding the co-occurrence of autophony and aural congestion were considered in the diagnosis of PET. Patients with positive results had their hearing evaluated via otoscopy, audiometry, and tympanometry.

## Results

**Figure 1:**
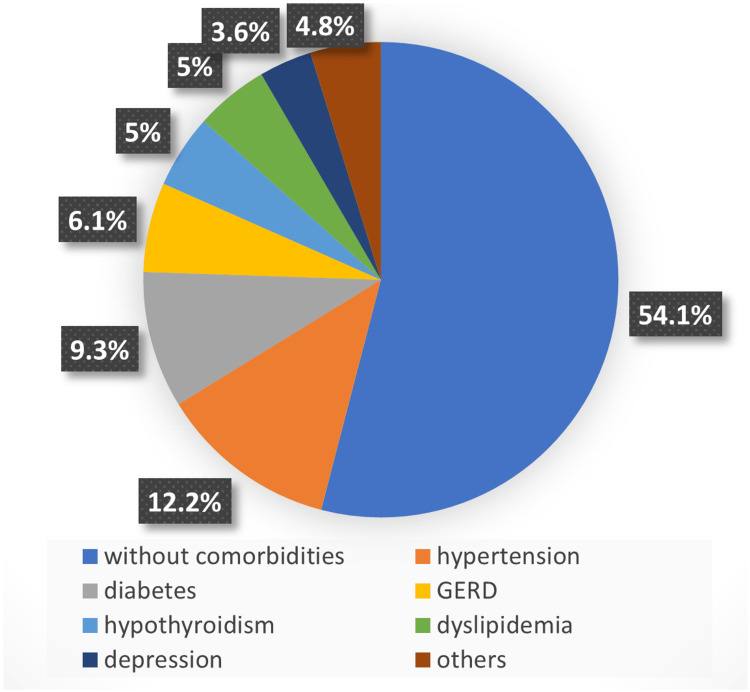
Comorbidities after bariatric surgery. GERD: gastroesophageal reflux disease

This study evaluates 450 patients who had undergone bariatric surgery over the period of 2022-2023, the patients’ age varied from 13 to 86 years old (mean = 37.25 years). Overall, 51.1% were females while 48.9% were males. About 46% of evaluated patients had at least one postoperative comorbidity, with the most prevalent being hypertension (12.2%), followed by diabetes mellitus (9.3%), and gastroesophageal reflux (6.1%) (Figure 1).

Among the 450 patients, 10.9% had a positive family history of ontological diseases while 89.1% did not. The preoperative BMI of evaluated patients ranged from 28 to 117 kg/m^2^ (mean = 46.07 kg/m^2^), while the postoperative BMI ranged from 16 to 100 kg/m^2^ (mean = 29.37 kg/m^2^). The total weight loss following bariatric surgery ranged from 4 to 130 kg (mean = 46.958 kg).

Regarding the otological complaints that presented in the postoperative period, autophony had the largest prevalence (19.6%), followed by aural fullness (3.3%). The concomitant complaints of autophony and aural fullness were present in 20.22% of the study population, compatible with the clinical diagnosis of PET dysfunction (Table 1).

**Table 1:** Frequency and percentage of patients per symptom in the postoperative period.

Symptoms	Yes	No
Autophony	88 (19.6%)	362 (80.4%)
Aural fullness	15 (3.3%)	435 (96.7%)
Feeling of listening to one’s own breath	13 (2.9%)	437 (97.1%)

Among patients with symptoms, the average preoperative BMI was 44.10 kg/m^2^, whereas those who were asymptomatic had an average preoperative BMI 46.66 kg/m^2^. There was no significant difference between the pre and postoperative BMI between patients with and without symptoms (Table 2).

**Table 2:** Comparison of patients with and without symptoms in relation to pre and postoperative body mass index (BMI).

	With symptoms	Without symptoms	Chi-square value	P-value
Preoperative BMI (kg/m^2^)	44.10	46.66	296.836	0.303
Postoperative BMI (kg/m^2^)	28.60	29.49	272.001	0.263

## Discussion

This study revealed a high prevalence (20.22%) of PET dysfunction among patients undergoing bariatric surgery. In the literature, few studies have related the presence of PET to significant weight loss, as in bariatric surgery. However, the medical literature describes neurotic anorexia in relation to PET in greater detail [10].

A 44-year-old lady who had undergone bariatric surgery presented with bilateral aural fullness and autophony in 2009, becoming the first case of PET to be published in the English-language literature [5]. Other studies have concluded that PET may be one of the surgical complications associated with obesity [11]. Munoz et al. conducted a similar study with 141 patients who had undergone bariatric surgery and estimated that 21.28% of their sample population had PET. The purpose of our investigation was to determine the prevalence of symptoms consistent with PET dysfunction due to acute weight loss following bariatric surgery. Bariatric surgery is recommended as a method for achieving short-term weight loss in morbidly obese patients and is a viable treatment option. In addition, obesity prevalence is on the rise in Saudi Arabia, resulting in a rapid expansion of the disease’s prevalence. Because all participants in this study had formal indications for surgery, there may be a significant impact on the function of the auditory tubules.

Interestingly, despite the presence of PET symptoms such as aural congestion and autophony, audiometry and tympanometry were not altered in our sample. Unfortunately, it is still difficult to make an objective diagnosis of auditory tubal dysfunction because symptoms are not always present at the time of evaluation (e.g., tympanometry) [9]. The patient’s medical history and current symptoms are used to determine the ultimate diagnosis [9].

We believe that the symptoms of PET dysfunction in our sample were related to the accelerated loss of adipocyte fatty tissue (Ostmann’s fat) that surrounds the cartilaginous portion of the eustachian tube. Similar findings were reported by other studies in similar conditions of weight loss, such as anorexia, puerperium, and restrictive alimentary diet [7,11].

Letti, et al. [12] evaluated a sample of eight patients who underwent a restrictive diet, losing an average of 15 kg in 45-65 days, and presented with tubal dysfunction. The loss of subcutaneous adipose fatty tissue predisposes the permeability of the auditory tubules, as noted by the authors of a study on the effects of a restrictive diet on patients with significant weight loss, poor nutrition, and deteriorating general health.

These patients are more likely to develop tubal dysfunction than the general population, as indicated by the previous studies, if they experience a sudden and significant weight loss. According to the literature, this is the first study conducted in the Middle East region. Additional research is required to interpret the association between the degree of weight loss and the severity of these symptoms, as well as long-term follow-ups with specific tests to evaluate the persistence and improvement of symptoms, along with their significance in the quality of life of the patient.

## Conclusions

Overall, 20.22% of the bariatric surgery patients in our study sample displayed symptoms consistent with patulous auditory tubal dysfunction. The preoperative and postoperative BMI of symptomatic and asymptomatic patients did not differ significantly. To improve the patient outcomes and satisfaction following bariatric surgery hearing symptoms associated with PET need to be included in bariatric surgery post operative follow-ups.
